# Capillary Transport of Miniature Soft Ribbons

**DOI:** 10.3390/mi10100684

**Published:** 2019-10-11

**Authors:** Bo Chang, Heng Liu, Robin H. A. Ras, Quan Zhou

**Affiliations:** 1 College of Mechanical and Electrical Engineering, Shaanxi University of Science and Technology, Xi’an 710021, China; Heng__Liu@163.com; 2 Department of Applied Physics, Aalto University, 02150 Espoo, Finland; robin.ras@aalto.fi; 3 Department of Electrical Engineering and Automation, Aalto University, 02150 Espoo, Finland; quan.zhou@aalto.fi

**Keywords:** micromanipulation, capillary force, capillary transportation, elasto-capillary, capillary gripper, hydrophilic/superhydrophobic patterned surfaces, soft ribbons

## Abstract

Manipulation of soft miniature devices is important in the construction of soft robots, wearable devices, and biomedical devices. However, transport of soft miniature devices is still a challenging task, and few studies has been conducted on the subject. This paper reports a droplet-based micromanipulation method for transporting miniature soft ribbons. We show that soft ribbons can be successfully picked up and released to the target location using water droplets. We analyze the forces involved during the process numerically and investigate the influence of the width of the ribbon on the deformation. We verify that the deformation of a soft ribbon caused by elasto-capillary phenomena can be calculated using a well-known equation for calculating the deflection of a cantilever beam. The experimental and theoretical results show that the deformability of a soft miniature device during manipulation depends on its width.

## 1. Introduction

Soft and stretchable miniature devices have attracted great interest for their applications in soft robotics [1,2], wearable devices [3], and biomedical devices [4]. For integrating components onto soft substrates, transfer printing has been widely used [5]. However, it is still very challenging to construct soft miniature devices, especially for three dimensional structures, because the dominant adhesion forces and deformation of the structures bring difficulties to basic manipulation tasks such as transport and placing. 

Manipulation of miniature soft objects has been previously demonstrated using elasto-capillary phenomena. Elasto-capillarity is the ability of capillary force to deform elastic material that has low elastic modulus and small characters size. The phenomena have been applied for wrinkling of an elastic membrane [6] and two-dimensional folding of a membrane [7,8]. Capillary forces may cause twisting and bending of two nearby micro-ribbons [9], clumping of wet hair [10], and bending and winding of microfibers around liquid droplet [11]. Elasto-capillary has also been applied for wrapping a droplet with an elastic sheet [12], folding of a polymer film by drop impact [13], and self-assembly of pyramid, spherical, cubic, and flower-shaped structures [14]. However, all the previous studies have focused on deformation of an elastic materials under the action of capillary force, and little research has been conducted on the transport and placing of soft structures. 

In this paper, we study how to use droplets to transport miniature soft ribbons. We use a droplet to pick up the ribbon and place it to a hydrophilic/superhydrophobic patterned surface. Depending on the width of the ribbon, two manipulation schemes are used. One is based on capillary interactions, and the other is based on elasto-capillary interactions. To understand the mechanisms, we simulate the shape of the liquid meniscus and model the capillary force during the transport. We demonstrate that soft ribbons made of polydimethylsiloxane (PDMS) can be successfully transported and placed on hydrophilic/superhydrophobic patterned surfaces.

## 2. Materials and Methods

### 2.1. Fabrication of Miniature Soft Ribbons

To fabricate miniature soft ribbons, superhydrophobic coating (Glaco Mirror Coat Zero, Soft99 Co., Osaka, Japan) is first spayed on a glass microscope slide (75 mm by 25 mm) serving as substrate and dried at room temperature. The superhydrophobic coating is applied to reduce surface adhesion. Then PDMS (Sylgard 184, Sigma-Aldrich, St. Louis, MO, USA) is spin coated on the glass slide at the room temperature with a rotation speed at 3000 rpm, resulting a PDMS film with a thickness of about 50 µm. Next, the PDMS film is baked at 120 °C for 20 min. The cured PDMS film is automatically cut into ribbons with a sharp blade mounted on a robotic cutting system. The cut ribbon is then manually picked up using tweezers. Ribbons with width in the range of 0.2 to 2 mm are fabricated. The manipulation surface is hydrophilic/superhydrophobic patterned surface. The fabrication procedure of hydrophilic/superhydrophobic patterned surface is similar to the process reported in a previous publication [15]. The fabrication was performed on a single side polished, 100 mm diameter, 500 µm thick silicon (100) wafer (Siegert Wafer GmbH, Aachen, Germany). Black silicon was formed in a cryogenic, inductively coupled plasma reactive ion etcher (ICP-RIE; Plasmalab System 100, Oxford Instruments, Abington, UK) for 7 min using experimentally optimized parameters: 40 sccm SF_6_ flow, 18 sccm O_2_ flow, 6 W forward power, 1000 W ICP power, −110 °C temperature, 10 mTorr pressure, with helium backside cooling. Standard photolithography was used to spin and pattern (2 µm) photoresist (AZ5214E, Siegert Wafer GmbH, Aachen, Germany) on top of the black silicon. Next, a thin film (around 100 nm) of fluorocarbon polymer coating was deposited, using CHF_3_ in a reactive ion etcher (Plasmalab80Plus, Oxford Instruments, Abington, UK). In the final step, the fluorocarbon polymer coating from pads was lifted-off in acetone for 20 min. The measured contact angles of the patterned surface are 50° and 170° on the hydrophilic site and the substrate, respectively.

### 2.2. Manipulation System

A manipulation system has been set up, adapted from previous experiments [16,17]. The system consists of a microscope, a needle dispensing system, a sample carrier, and a water mist dispenser. The microscope (Edmund/VZM1000i, Edmund Optics, Barrington, NJ, USA) is used to record the transport and placing process. The needle dispensing system (Cavro Centris, Tecan Group Ltd., Mendendorf, Switzerland) is used to dispense water droplet on the sample and as a manipulation tool. The outer and inner diameter of the needle are 0.41 mm and 0.2 mm respectively. The sample carrier is mounted on three motorized stages (M-111.1DG, M-122.2DD, and M-404.8PD by Physik Instrument, Kasru, Germany), which allow the movement of the sample in the XYZ directions with micrometer precision. The water mist dispenser is a modified air humidifier (Bionaire Ultrasonic Compact BU1300W-I by Bionaire, Las Vegas, NV, USA) where 12 mm silicon tube was attached to the outlet of the humidifier that can dispense water mist to the desired area. 

### 2.3. Transport Schemes

**Figure 1:**
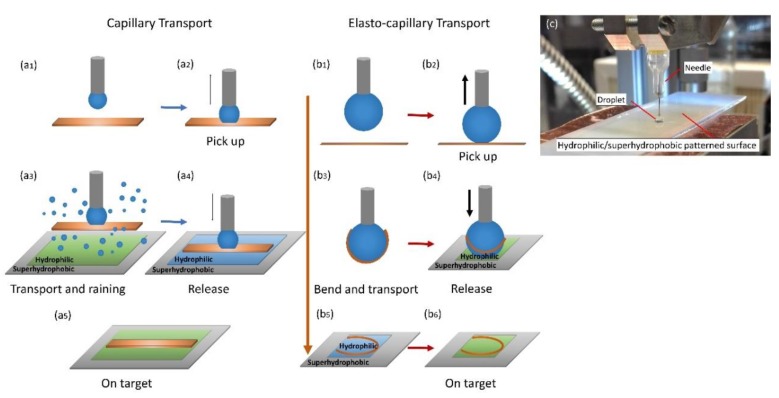
Schematic and system setup for transport of miniature elastic ribbons using capillary transport and elasto-capillary transport scheme. (**a_1_**) and (**b_1_**) A droplet is produced with a needle; (**a_2_**) and (**b_2_**) the needle with the droplet picks up the ribbon; (**a_3_**) the ribbon is transported to the hydrophilic/superhydrophobic patterned surface and water mist is introduced; (**b_3_**,**b_4_**) the ribbon bends and is transported to a hydrophilic/superhydrophobic patterned surface; (**a_4_**) and (**b_5_**) the water film is formed on the hydrophilic site and the ribbon is released on the hydrophilic site; (**a_5_**) and (**b_6_**) the ribbon is on target; (**c**) system setup for transport of elastic ribbon.

We studied two transportation schemes—one is based on mainly capillary adhesion, and the other is based on elasto-capillarity. Both schemes employ droplets to achieve the transport and placing tasks. For the ribbons with width similar to the size of the manipulation droplet, the ribbon barely bends when contacting the manipulation droplet, as shown in Figure 1a. To transport the ribbon, first, the needle dispenses water creating a pendent droplet under the needle (Figure 1a_1_). Then, the needle with the droplet moves downward and contacts the ribbon, forming a liquid meniscus. If the capillary force of the meniscus overcomes the surface adhesion between the ribbon and the substrate, the ribbon can be picked up by the needle (Figure 1a_2_). After that, the ribbon is transported to a target hydrophilic/superhydrophobic patterned surface, water mist is introduced on the patterned surface, and a meniscus is formed on the hydrophilic site (Figure 1a_3_,a_4_). If the capillary force between the meniscus on the hydrophilic site and the lower surface of the ribbon is larger than the capillary force between the meniscus below the needle and the top surface of the ribbon, the ribbon is released on the target location (Figure 1a_4_,a_5_). For the ribbons with a width much smaller than the size of the manipulation droplet, the ribbon bends when contacting the manipulation droplet, as shown in Figure 1b. The manipulation procedure is similar to the previous one, but the interactions are different. First, the needle dispenses a water droplet (Figure 1b_1_). Then, the ribbon is picked up by the needle, and the ribbon wraps the droplet (Figure 1b_3_). The ribbon is then transported to a hydrophilic/superhydrophobic patterned surface (Figure 1b_4_). When the droplet with the ribbon is in contact with the target hydrophilic site, the droplet forms a flat meniscus on the site, and the ribbon is consequently released (Figure 1b_5_). After the water is vaporized, the ribbon is left on the target site (Figure 1b_6_).

## 3. Results

### 3.1. Capillary Transport

**Figure 2:**
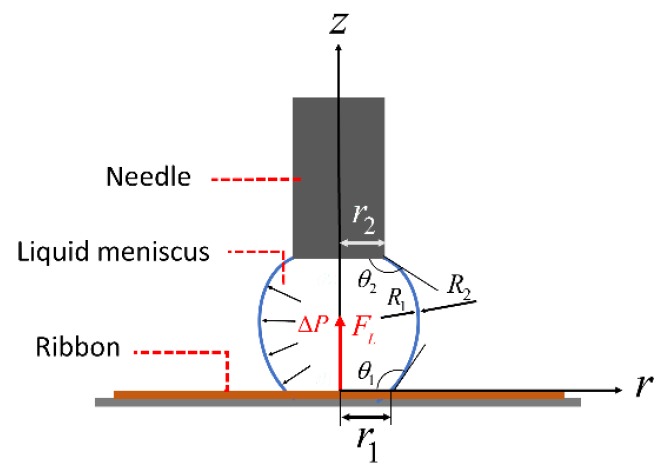
Force illustration of lifting a ribbon.

When the needle contacts the soft ribbon, a liquid meniscus is formed between the needle and the elastic ribbon, as shown in Figure 2. The ribbon can be picked up by the capillary force between the liquid meniscus and the ribbon. In such a case, we use ribbons with widths similar to the size of the manipulation droplet. Consequently, the radius of the contact area between the meniscus and the ribbon $r_{1}$ is usually smaller than the width of the ribbon, so we can estimate the capillary lifting force $F_{L}$ using an axial symmetric model. The capillary lifting force $F_{L}$ consists of two components, the force caused by the pressure difference between the inside and the outside of the liquid meniscus $F_{p}$ and the vertical component of the surface tension $F_{t}$. The capillary lifting force can be expressed as

(1) $$F_{L}=F_{p}+F_{t}=\pi r_{1}{}^{2}\Delta P+2\pi r_{1}\gamma \sin(\theta_{1})$$

where γ is the surface tension of the liquid, $\theta_{1}$ is the water contact angle on the ribbon, $r_{1}$ is the radius of the contact area, and ΔP is the pressure difference determined from Young-Laplace equation.

(2) $$\Delta P=\gamma H=\gamma \left(\frac{1}{R_{1}}+\frac{1}{R_{2}}\right)$$

where H is the mean curvature of the meniscus, $R_{1}$ and $\;R_{2}$ are the principal radii of curvature. Based on the geometry of the meniscus, the mean curvature H can be expressed with respect to the coordinate of the meniscus r [18].

(3) $$\frac{1}{R_{1}}+\frac{1}{R_{2}}=\frac{r^{\prime \prime }}{{(1+r^{\prime }}^{2}{)}^{\frac{3}{2}}}-\frac{1}{r{(1+r^{\prime }}^{2}{)}^{\frac{1}{2}}}$$

Therefore, the capillary lifting force can be calculated if we know the exact shape of the meniscus. To estimate the shape of the meniscus, we developed a double iteration algorithm reported in an early publication [19]. The algorithm starts with selecting a random starting point on the meniscus and setting the mean curvature, then the differential equation (Equation (3)) is solved, and the shape of the meniscus is obtained. After that, the contact angle on the ribbon is obtained and compared with the given contact angle. If the difference is less than the defined error interval, then the volume of the liquid is calculated. Otherwise, the mean curvature is reset. If the contact angle falls within the error interval, the volume of the liquid is calculated and compared with the given volume. If it is within the error interval, then the capillary lifting force is computed using Equation (1), otherwise the starting point is reselected.

**Figure 3:**
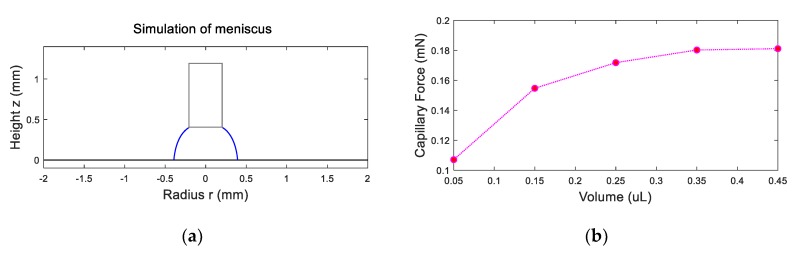
Simulation of meniscus and capillary lifting force analysis: (**a**) simulation of water meniscus of 0.15 μL with double iteration method; (**b**) capillary lifting force as the function of volume of the meniscus.

Figure 3a shows the simulation of a water meniscus (0.15 μL) between a needle and a ribbon. Given the water apparent contact angle being 90° and 150° on the ribbon and the needle, respectively, and the distance between the needle and the ribbon 0.4 mm, the calculated capillary lifting force should be 0.15 mN. Figure 3b shows the relationship between the capillary lifting force and the volume of meniscus. The simulation results indicate that the capillary lifting force increases as the volume of the meniscus increases. 

To pick up the ribbon, the capillary lifting force $F_{L}$ should be greater than the sum of the adhesion between the ribbon and the substrate $F_{A}$ and the gravity of the ribbon G. Given the length of the elastic ribbon *L* = 5 mm, the width *w* = 2 mm, the thickness *h* = 50 μm, the mass density of PDMS ρ = 965 kg/m^3^, and the gravity of the ribbon can be estimated using

(4) $$G=\rho Lhwg=4.73\times 10^{-3}\;\mathrm{mN}$$

The adhesion force by surface unit between two solid surfaces mainly include van der Waals force, which can be expressed as [20]

(5) $$F_{A}=\frac{A}{6\pi z^{3}}$$

where A is the Hamaker constant, and z is the distance between the ribbon and the substrate, which can be considered in the order of nanometers [20]. The calculated Hamaker constant between Nylon and solid PDMS is in the order of $5.4\times 10^{-20}\;J$ [21], so the adhesion force $F_{A}$ should be in the order of micronewtons at the same level of gravity. Both forces jointly contribute less than 10% of the capillary lifting force $F_{L}$ (0.1–0.2 mN), thus the ribbon should be picked-up easily. 

**Figure 4:**
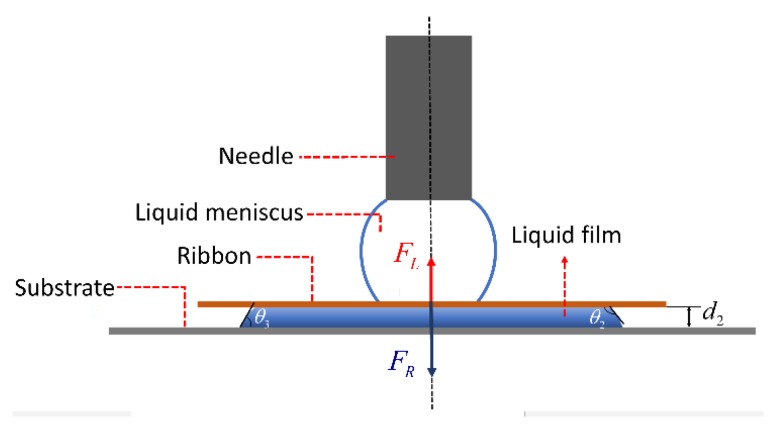
Force illustration of releasing a ribbon.

For releasing of the ribbon, water mist is introduced on the hydrophilic substrate and a water meniscus is formed between the ribbon and the substrate as shown in Figure 4. Given the water contact angle on the ribbon and the hydrophilic substrate $\theta_{2}=90{}^{\circ}$, $\theta_{3}=30{}^{\circ}$, the height of the meniscus $d_{2}=0.05\;\mathrm{mm}$, the volume of the liquid 4 μL, the releasing force $F_{R}$ should be around 114 mN, which is three orders of magnitudes greater than the capillary lifting force $F_{L}$ (0.15 mN). Therefore, the ribbon releases rather deterministically.

**Figure 5:**
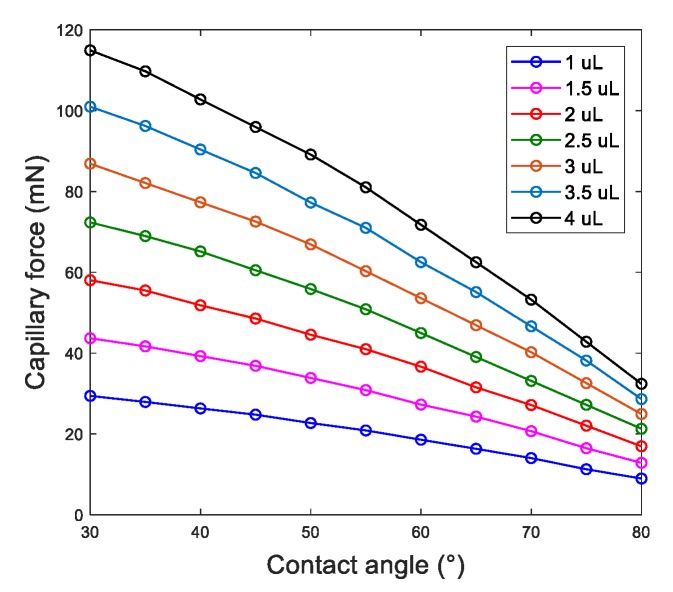
Force illustration of releasing a ribbon.

Figure 5 shows the capillary releasing force as the function of the volume of the meniscus and the contact angle on the substrate, where *x*-axis represents the contact angle on the substrate, and *z*-axis represents the capillary releasing force. The releasing force increases as the volume of the meniscus increases and the contact angle on the substrate decreases. The simulation indicates that the releasing mainly depends on the volume of the liquid film between the ribbon and the hydrophilic site and the water contact angle on the hydrophilic site. 

**Figure 6:**
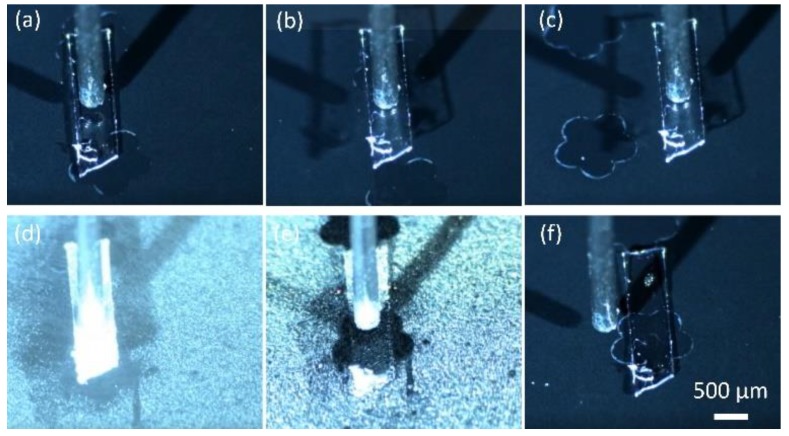
Capillary transport of miniature elastic ribbon. (**a**) a needle is above a ribbon; (**b**) the ribbon is picked up by the needle; (**c**) the ribbon is transported to the target location; (**d**) water mist is introduced; (**e**) the ribbon is released on the target site; (**f**) water droplets evaporate. The images are snapshots from the Supplementary Video S1.

Figure 6 shows the sequences of picking up and releasing of a ribbon. First, the ribbon is moved under the needle (Figure 6a), and then a droplet is produced by the needle and the ribbon is picked up (Figure 6b). Next, the ribbon is transported to the target site, and water mist is introduced to the site (Figure 6c,d). Finally, the ribbon is released on the target, and the droplets evaporate (Figure 6e,f). 

### 3.2. Elasto-Capillary Transport

**Figure 7:**
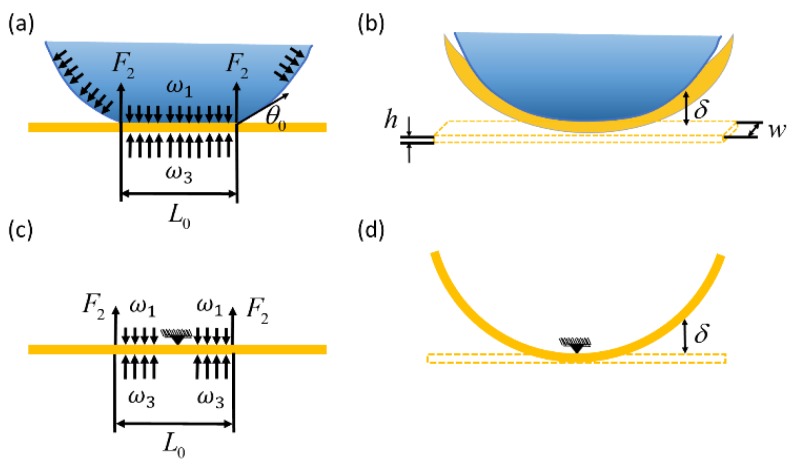
Electro-capillary transport scheme: (**a**,**b**) force illustration; (**c**,**d**) deformation model of a ribbon as the deflection of a cantilever beam.

Based on the scaling laws, capillary force is proportional to length l, elastic forces are proportional to $l^{2},$ and body forces such as gravity is proportional to $l^{3}$. When all the dimensions scale down, capillary force decreases the slowest compared to elastic forces and gravity force. Therefore, in micro scale, when a drop is in contact with an elastic ribbon and the width of the ribbon is much smaller than the size of the droplet, the capillary force will eventually overcome the elastic force and bend the elastic ribbon, as shown in Figure 7.

**Figure 8:**
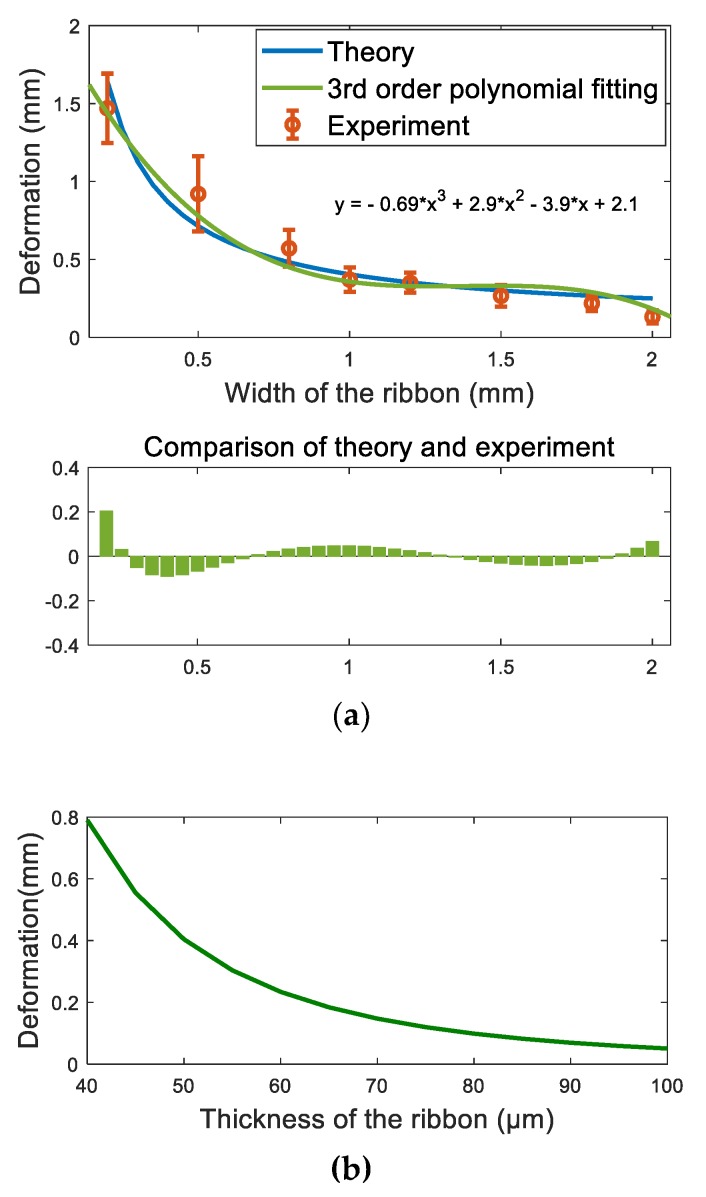
Influence of the width and thickness on the deformation of the ribbon. (**a**) deformation as the function of the width and comparison of the theoretical estimation and experiments; (**b**) deformation as the function of the thickness.

The bending of the ribbon can be modelled as the deflection of a cantilever beam given that the droplet is dispensed in the middle of the ribbon. As shown in Figure 7, $L_{0}$ is the contact length of a droplet with the elastic ribbon, w and h are width and thickness respectively. δ is the deformation of the ribbon, which is defined as the distance between the middle of the ribbon and the water–ribbon contact line. The bending force acting on the elastic ribbon consists of three parts, one is the uniformly distributed load $\omega_{1}$ caused by Laplace pressure $\Delta P=\gamma \left(\frac{1}{R_{1}}+\frac{1}{R_{2}}\right)$, and the second part is the concentrated load $F_{2}$, which is the vertical component of the surface tension along the width of the ribbon and can be calculated as $w\gamma \sin(\theta_{0})$. The last part is the uniformly distributed load $\omega_{3}=\gamma \sin(\theta_{1})$ which is also the vertical component of the surface tension along the contact length $L_{0}$. Therefore, the deformation can be calculated using Equation (6).

(6) $$\delta =\frac{\omega_{3}{\left(\frac{L_{0}}{2}\right)}^{4}}{8EI}+\frac{F_{2}{\left(\frac{L_{0}}{2}\right)}^{3}}{3EI}-\frac{\omega_{1}{\left(\frac{L_{0}}{2}\right)}^{4}}{8EI}$$

where E is Young’s modulus of the elastic ribbon, γ is the surface tension, I is the moment of inertia of an elastic ribbon, which can be expressed as $wh^{3}/12$, $\theta_{0}$, is the water contact angle on the ribbon. The blue curve shown in Figure 8a represents the deformation of the ribbon as the function of the width and thickness, which is calculated using Equation (6), given the water contact angle on the ribbon $\theta_{0}=90{}^{\circ}$, Young’s modulus of PDMS E = 3.2 MPa, the width of ribbon w=0.2–2 mm, and the thickness of the ribbon h=40–100 µm. The deformation of the ribbon is clearly affected by both the width and thickness. The deformation of the ribbon decreases as the width of the ribbon and the thickness increase.

**Figure 9:**
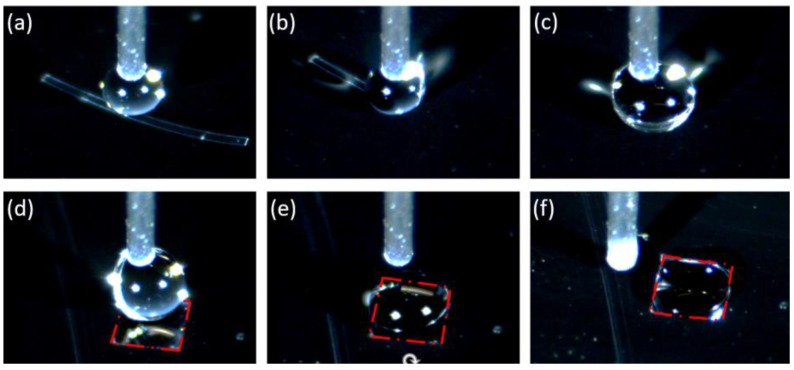
Elasto-capillary transport of miniature elastic ribbon. (**a**) a droplet is produced with a needle; (**b**) the needle with the droplet picks up the ribbon; (**c**,**d**) the ribbon bends and is transported to the hydrophilic site; (**e**,**f**) the droplet is in contact with the hydrophilic site and the ribbon is released on the target. The images are snapshots from the Supplementary Video S1.

Figure 9 shows the sequences of picking up and releasing of a 0.2 mm wide ribbon based on elasto-capillary phenomenon. First, the ribbon is moved below the needle (Figure 9a), and then a droplet is produced by the needle. Next, the ribbon bends and is picked up by the needle (Figure 9b,c). After that, the ribbon is transported to a hydrophilic target site, which is highlighted with red dot lines (Figure 9d). Finally, and water droplet is in contact with the hydrophilic site, forming a liquid film, and the ribbon is released on the target (Figure 9e,f).

**Figure 10:**
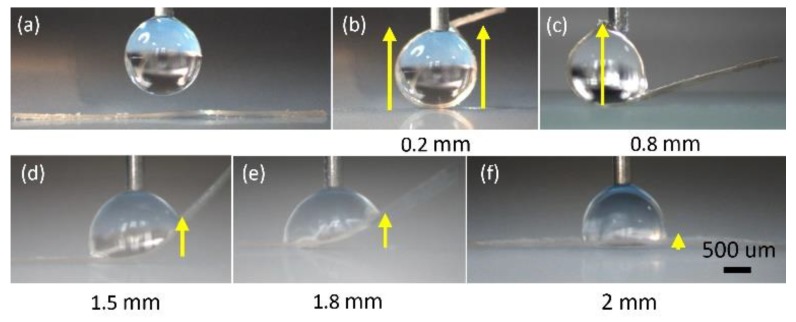
Deformation of the ribbon with different width. (**a**) A 1 µL droplet is dispensed at the middle of the ribbon; the ribbon with width of (**b**) 0.2 mm, (**c**) 0.8 mm, (**d**) 1.5 mm, (**e**) 1.8 mm, and (**f**) 2 mm bend. The deformation of the ribbon is highlighted with yellow arrow line.

To verify the theoretical estimations of the deformation as the function of the width of the ribbon shown in Figure 9a, a series of tests were carried out to study the influence of the width of the ribbon on the deformation. In the tests, the length of the ribbon was kept same as 5 mm, the thickness of the ribbon is 50 µm, and the contact angle of the water on the PDMS is 90°. Ribbons with widths of 0.2 mm, 0.5 mm, 0.8 mm, 1.0 mm, 1.2 mm, 1.5 mm, 1.8 mm, and 2 mm were tested. The same amount of the droplet (1 µL) was dispensed at the middle of the ribbon for each test, and each test was repeated five times. The deformation was measured as the distance between the middle of the ribbon and the water-ribbon contact line. The experimental results are shown in Figure 8a and compared with the theoretical estimations. The theoretical estimations are given based on Equation (6). The orange circles with error bar represent the experimental data, which consists of mean of five repetitions with standard derivation. The green curve is the third order polynomial fitting of the experimental data and the blue curve represents the theoretical estimations. Both experimental results and theoretical estimations show that the ribbons with widths from 0.2 mm to 2 mm all bend, and as the width of the ribbon increases, the ribbons deform less. The experimental data matches the theoretical estimations well, and both indicate that the ribbon with smaller width produces larger deformation. Equation (6) is a well-known equation for calculating the deflection of a cantilever beam in mechanical design, our study verifies that Equation (6) is also valid for calculating deformation of soft ribbons, which has not been reported before. Figure 10 shows examples of the deformation of the ribbon as the width of the ribbon increases from 0.2 mm to 2 mm. First, a droplet is dispensed at the middle of the ribbon by a needle (Figure 10a). Then, a meniscus is formed, and the ribbons with different widths all bend (Figure 10b–f).

## 4. Conclusions

This paper studied a manipulation method for transporting miniature soft ribbons using droplets. Two transportation schemes have been demonstrated—one is mainly based on the capillary force, and another is based on the elasto-capillary phenomenon. We demonstrated that both methods are effective for soft ribbon transportation and placing, and more specifically, the capillary transport scheme is suitable for wide ribbons that are similar to the size of the manipulation droplet and barely deform during the transportation process. The elasto-capillary transportation scheme can be used to handle very slim ribbons. We further investigated the influence of the width of the ribbon on deformation and verified (both theoretically and experimentally) that the deformation of the ribbon decreases as the width of the ribbon increases. Additionally, we clarify that the deformation of a soft ribbon caused by elasto-capillary phenomena can be calculated using a well-known equation for calculating the deflection of a cantilever beam. Those results indicate a soft ribbon can be treated as a rigid object when the diameter of the manipulation droplet is smaller than the width of the ribbon, and if the diameter of the manipulation droplet is larger than the width of the ribbon, the ribbon will always bend, and how much it deforms depends on its width. This work provides valuable information to construct soft devices, and in future work, we will carry out further studies on more complex structures using the capillary-based micromanipulation method.

## Supplementary Materials

The following are available online at https://www.mdpi.com/2072-666X/10/10/684/s1, Video S1: Capillary Transport of Miniature Soft Ribbons.
